# Our Environmental Value Orientations Influence How We Respond to Climate Change

**DOI:** 10.3389/fpsyg.2019.00938

**Published:** 2019-06-18

**Authors:** N. A. Marshall, L. Thiault, A. Beeden, R. Beeden, C. Benham, M. I. Curnock, A. Diedrich, G. G. Gurney, L. Jones, P. A. Marshall, N. Nakamura, P. Pert

**Affiliations:** ^1^CSIRO Land and Water, CSIRO Oceans and Atmosphere, Townsville City, QLD, Australia; ^2^College of Science and Engineering, James Cook University, Townsville City, QLD, Australia; ^3^National Center for Scientific Research, PSL Université Paris, CRIOBE, USR 3278 CNRS-EPHE-UPVD, Maison des Océans, Paris, France; ^4^Laboratoire d’Excellence CORAIL, Moorea, French Polynesia; ^5^Queensland Health, Townsville City, QLD, Australia; ^6^Great Barrier Reef Marine Park Authority, Townsville City, QLD, Australia; ^7^ARC Centre of Excellence, James Cook University, Townsville City, QLD, Australia; ^8^The London School of Economics and Political Science, London, United Kingdom; ^9^Reef Ecologic, Townsville City, QLD, Australia

**Keywords:** environmental behavior, cultural ecosystem services, ecological grief, natural resource management, communication, Great Barrier Reef, Australia, coastal communities

## Abstract

People variably respond to global change in their beliefs, behaviors, and grief (associated with losses incurred). People that are less likely to believe in climate change, adopt pro-environmental behaviors, or report ecological grief are assumed to have different psycho-cultural orientations, and do not perceive changes in environmental condition or any impact upon themselves. We test these assumptions within the context of the Great Barrier Reef (GBR), a region currently experiencing significant climate change impacts in the form of coral reef bleaching and increasingly severe cyclones. We develop knowledge of environmental cultural services with the Environmental Schwartz Value Survey (ESVS) into four human value orientations that can explain individuals’ environmental beliefs and behaviors: biospheric (i.e., concern for environment), altruistic (i.e., concern for others, and intrinsic values), egoistic (i.e., concern for personal resources) and hedonic values (i.e., concern for pleasure, comfort, esthetic, and spirituality). Using face-to-face quantitative survey techniques, where 1,934 residents were asked to agree or disagree with a range of statements on a scale of 1–10, we investigate people’s (i) environmental values and value orientations, (ii) perceptions of environmental condition, and (iii) perceptions of impact on self. We show how they relate to the following climate change responses; (i) beliefs at a global and local scale, (ii) participation in pro-environmental behaviors, and (iii) levels of grief associated with ecological change, as measured by respective single survey questions. Results suggest that biospheric and altruistic values influenced all climate change responses. Egoistic values were only influential on grief responses. Perception of environmental change was important in influencing beliefs and grief, and perceptions of impact on self were only important in influencing beliefs. These results suggest that environmental managers could use people’s environmental value orientations to more effectively influence climate change responses toward environmental stewardship and sustainability. Communications that target or encourage altruism (through understanding and empathy), biospherism (through information on climate change impacts on the environment), and egoism (through emphasizing the benefits, health and wellbeing derived from a natural resource in good condition), could work.

## Introduction

Climate change perceptions and beliefs are changing, globally. Extreme events such as flooding, and slow, relentless chronic events such as drought mean that people are experiencing, first hand, climate change impacts on the special places within which they live and work (Devine-Wright, 2013; Heimann and Mallick, 2016; Nicolosi and Corbett, 2018). Increasingly, it is recognized that people’s experiences with these local scale impacts provides an important impetus to review and update their global climate change beliefs and risk perceptions (Myers et al., 2012; Hansen and Cramer, 2015; Lee et al., 2015). Specifically, people are beginning to connect local events to human behaviors and causes at a global level, despite massive resistance and denial to this concept (van der Linden, 2015). However, changes in perceptions and beliefs have been extremely slow to develop, and whilst a momentum is gradually growing, acceptance that climate change is a human-caused phenomenon, and that behavior change is urgently required, still has far to go. For example, in New Zealand, Milfont et al. (2015) profiled over 6,000 residents as: those who believe in the reality of climate change and its human cause (53%), those undecided (30%), the complete skeptics (10%), and those who believe the climate is changing but is not caused by human activity (7%) (Milfont et al., 2015).

Most typically, acceptance of climate change is higher among younger females with pro-environmental or longer-term outlooks and characterized by significantly lower levels of right wing authoritarian and social dominance beliefs (Joireman and Liu, 2014; Devine-Wright et al., 2015). At least within developed county contexts, a very clear “conservative white male” effect has concurrently emerged, which doubts the science of human-caused climate change (Devine-Wright et al., 2015; Milfont et al., 2015). Nonetheless, around the world, even in industries in which it has been detrimental to acknowledge human driven climate change, a majority of people are now in agreement. In 2012, for example, Rogers et al. (2012a) reported that most rural landholders in south-eastern Australia were no longer climate change “deniers,” with 70% agreeing with the statement, “The climate is changing and that human activity is a major influence” (Rogers et al., 2012a).

Concurrent with growing awareness of climate change, researchers are also reporting increasing levels of concern (Gatersleben et al., 2010). Well-documented responses to perceived catastrophic and large scale impacts and threats include a sense of disempowerment and helplessness (O’Neill and Nicholson-Cole, 2009), and even anxiety and depression (Searle and Gow, 2010). Walker et al. (2015), having surveyed over 5,000 people, reported that about one-third of Australians believed environmental quality was worsening as a result of climate change and felt angry about it (Walker et al., 2015). Marshall et al. (2019) have reported that around half of coastal residents, tourists and tourist operators, and almost one quarter of commercial fishers in the catchments of the Great Barrier Reef, expressed significant grief after reports of the degrading state of the Great Barrier Reef (GBR) resulting from climate change-related events (Marshall et al., 2019). Ecological grief is a phenomenon that needs to be better acknowledged in order to better understand the range of responses that occur in response to understanding what the consequences of climate change mean (Barnett et al., 2016). Ecological grief describes the emotional suffering associated with losses to valued species, ecosystems and landscapes that occur as a result of climate change (Benham, 2016; Bartual, 2017; Cunsolo and Ellis, 2018). Indeed, researchers are considering a new science, a science of loss, to document and make sense of the feelings associated with the devastation that climate change is causing (Barnett et al., 2016).

The extent to which people recognize that the environment is degrading as a result of climate change related events, and the extent to which people expect to be impacted, are also likely to be an important influence on people’s perceptions of, and response to, climate change (Marshall et al., 2013). The premise is, if people recognize changes in the environment, then they are better placed to respond. If people are worried that the environmental changes will impact upon them either physically, financially, socially, or emotionally, then they may be more likely to develop their climate change awareness. Marshall et al. (2013) found that people that had limited climate change awareness appeared to be restricted in their ability to manage the risks associated with climate change, plan for change or be interested in undertaking behavior change.

Pro-environmental behavior is essential both within urban and rural settings. In the United Kingdom for example, 40% of carbon emissions are attributed to household and transport behavior (Gatersleben et al., 2010; Fudge and Peters, 2011; Poortinga et al., 2012). Similarly, agricultural practices are major contributors to greenhouse gas emissions (Fleming and Vanclay, 2011). Yet, whilst people are beginning to acknowledge the need for behavior change in order to both mitigate and adapt to the effects of climate change, there appears to be an obvious lag in observing any real behavior change (Fudge and Peters, 2011; Ortega-Egea et al., 2014; Wynveen and Sutton, 2017). In the United Kingdom, Gatersleben et al. (2010) found that on the one hand, quite a number of people expressed both high levels of concern for climate change, but on the other hand, reported high levels of materialism, suggesting that people have not as yet articulated how they want to respond to the reality of climate change. That is, whilst shifting societal attitudes toward accepting climate as a major problem is critical for climate awareness and for changing behavior toward both mitigation and adaptation actions, behavior change has been slow. This may be because climate change is not necessarily seen as “interesting” even by highly engaged people (Howell, 2013).

Influencing pro-environmental behavior is becoming particularly important as the impacts of climate change worsen (Wynveen and Sutton, 2017). Belief in, and knowledge of, climate change have been linked to the adoption of more strategic adaptations in some resource-dependent industries (Rogers et al., 2012b; Marshall et al., 2013), and work inspired from Stern’s value-belief-norm (VBN) theory of environmentalism suggests that perceiving adverse effects from global warming could promote mitigation behaviors (Chen, 2015). However, a growing literature is suggesting that concern about the environment should not be the main message to communicate to people in order to influence their behavior, as it may not be a primary motivation for change (Cook and Ma, 2014). Researchers are suggesting that the key catalysts for change encompass factors such as social justice, community, frugality, personal integrity, health, and beliefs in self-efficacy (Bostrom et al., 2013; Howell, 2013), in addition to the way in which climate change messaging is framed to reflect cultural values (Corner et al., 2014; Baldwin and Lammers, 2016). It appears that having a positive attitude is important, believing that the climate has been changing over the previous 30 years, and having a stronger belief in human activities influencing the climate (Cook and Ma, 2014). Women are more likely than men to adopt pro-environmental behaviors (Howell, 2013; Joireman and Liu, 2014), and so are people in societies characterized by higher levels of trust, belief in internal control, and with higher levels of individualism and “looseness” (Kalamas et al., 2014; Tam and Chan, 2017). More recent thinking suggests that the decision to adopt appropriate pro-environmental behaviors will reflect some general psychological orientations, or values, that are culturally patterned (Fielding and Hornsey, 2016; Tam and Chan, 2017).

Our purpose is to explore and add to the developing momentum of knowledge suggesting that psycho-cultural factors or value orientations shape the varied responses to climate change (Price et al., 2014; Leombruni, 2015; van der Linden, 2015; Bouman et al., 2018). We merge psycho-cultural perspectives with cultural ecosystem services through the framing of the Environmental Schwartz Value Survey (ESVS) (Bouman et al., 2018), in which four human value orientations are used to explain individuals’ environmental beliefs and behaviors: biospheric (i.e., concern for environment), altruistic (i.e., concern for others), egoistic (i.e., concern for personal resources) and hedonic values (i.e., concern for pleasure and comfort). Other authors have tested for reliability and validity across a range of studies and suggested that the four categories offer a useful approach to assessing values, with expected validity issues, such as women value benevolence more than men (Lindeman and Verkasalo, 2005). Drawing on Marshall et al. (2018)’s framework of human-environment cultural values and that of Hicks et al. (2015), who independently provided value clusters for cultural ecosystem services, we examine different meanings or values that people hold for the GBR and organize them according to the ESVS, as such (Hicks et al., 2015; Marshall et al., 2018):

(1) Biospheric (appreciation of biodiversity, and scientific heritage benefits)
(2) Altruistic (appreciation of intrinsic values, and Traditional Owner heritage),
(3) Egoistic (appreciation of health benefits, wisdom and way of life, economic values, wellbeing, and lifestyle)
(4) Hedonic (appreciation of spiritual, artistic, and esthetic opportunities)

Our aims were to explore the influence of these value orientations on each of the following climate change responses: (i) global and local climate change beliefs, (ii) level of ecological grief in response to climate change related environmental degradation, and (iii) pro-environmental behaviors that are climate change specific. In doing so, we control for both perceptions of environmental impact (personal experience) and perceptions of the impact on self. As such, we expected to develop important insights into how people respond to climate change and the value orientations that influence their response. Specifically, we explore the influence of these psycho-cultural factors on how people respond to climate change and test each of the following hypotheses:

(1) Reef Grief is affected by value orientations
(2) Climate change beliefs are affected by value orientations
(3) Pro-environmental behaviors are affected by value orientations.

## Case Study Context

We examine these hypotheses within the context of the GBR, a region currently experiencing significant ecological, economic and social change. The GBR is the largest coral reef ecosystem on Earth, spanning 2,300 km along the east coast of Queensland, Australia. It is one of the most inspiring landscapes within Australia (Marshall et al., 2016; Goldberg J.A. et al., 2018) and is an important part of the identity of people not only residing in Queensland but also in Australia and overseas (Gurney et al., 2017). It supports a community of nearly 800,000 people, and produces around $6.4 billion per year of economic activity (Deloitte Access Economics, 2017). The GBR is a vital contribution to the wellbeing of the local people, as well as for Australians more broadly (Larson et al., 2013, 2015). Recent surveys have documented the rich and diverse relationship that local residents, Australians, tourists, commercial fishers and tourism operators have with the GBR including use, attitudes, perceptions of threats, experiences, values, aspirations, and levels of satisfaction (Gurney et al., 2017; Marshall et al., 2017). For example, 90% of local residents in the region felt that the GBR had outstanding beauty, and were proud of its World Heritage Area status.

Following a spate of severe and cumulative regional-scale impacts related to climate change, including tropical cyclones, and mass coral bleaching (in both 2016 and 2017), and an ongoing outbreak of coral-eating crown of thorns starfish, recent ecological monitoring suggests that the proportion of live coral coverage across all regions of the World Heritage Area have undergone a steep decline, to an extent not observed in the historical record (AIMS Long-Term Monitoring Program 2018, available at https://www.aims.gov.au/reef-monitoring/gbr-condition-summary-2017-2018). Accordingly, there has been intense media coverage surrounding the events, and sometimes misleading information around climate change threats and impacts, where the “normal” background variability in extreme climate impacts such as cyclones has made it problematic to determine whether an individual event (such as a cyclone, bush fire, drought even coral bleaching) is directly attributable to climate change (Lankester et al., 2015).

Recent research has highlighted the high level of ecological grief, or “reef grief” that is currently thus being experienced by local people (Marshall et al., 2019). However, and importantly, the impacts of global climate change on the GBR are difficult to observe at the local level given the considerable spatial and temporal variability in the patterns of impacts that occur. Impacts cannot be personally experienced across large spatial scales, unless (potentially), viewed aerially. Hence, many local people have not directly observed the effects of climate change on the Great Barrier Reef, and instead must depend on various media sources for information and knowledge of current state and status of the GBR (Lankester et al., 2015).

## Materials and Methods

Survey data were obtained from the Social and Economic Long Term Monitoring Program (SELTMP) for the GBR (Marshall et al., 2016). Data are publically available at www.csiro.au/seltmp.

### Survey Design

**FIGURE 1:**
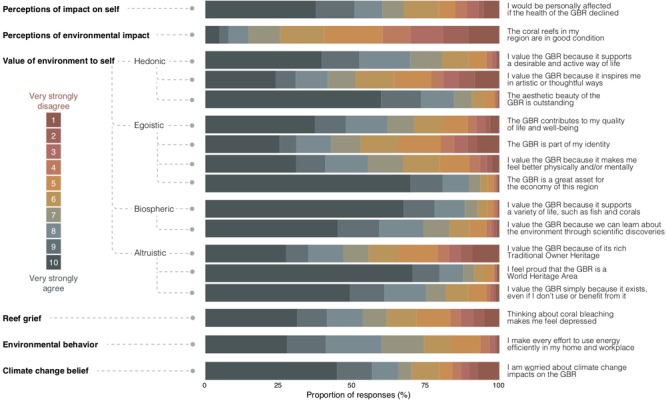
Participant responses to the survey questions designed to capture each value orientation and each climate change response. Survey participants were asked to agree or disagree with each survey statement on a ten-point scale where a rating of 1 represented “very strongly disagree” and 10 represented, “very strongly agree” (*n* = 1923).

A single survey statement was used to assess each of (i) perceptions of environmental impact, (ii) perceptions of impact on self, and (iii) reef grief (Figure 1). These questions are presented in Figure 1. Whilst we recognize that a single survey statement is unlikely to adequately represent the complexity of each of the concepts, we were practically limited, and suggest that any results are indicative of each concept only. Twelve survey questions were used to understand what values were important to people, and were categorized according to the ESVS. These survey questions are also presented in Figure 1. Survey participants were asked to agree or disagree with each survey statement on a ten-point scale where a rating of 1 represented “very strongly disagree” and 10 represented, “very strongly agree.” A weighted mean was developed for each value category using a principal component analysis, where the survey responses were forced into the one factor score, after internal reliability was confirmed. Pro-environmental behaviors were measured by asking people to agree with each of four statements about environmental behavior. Given that all behaviors were correlated (Pearson correlations = 0.223^∗∗^, 0.288^∗∗^, and 0.398^∗∗^), we only used the following statement to represent all pro-environmental behaviors; “I make every effort to use energy efficiently in my home and workplace.” We also quantified age and gender. Global climate change beliefs were elicited through asking participants to describe which of the given statements best reflected their views on climate change (Figure 1). Given that global climate change beliefs were significantly correlated with local climate change beliefs (0.285^∗∗^), we only used local climate change beliefs in analyses.

### Survey Administration

Interviews were undertaken on an Apple mini-iPad loaded with an iSurvey application in public places such as parks, shopping centers, market places, airports, marinas, sporting areas, festivals, information centers, museums, jetties, caravan parks, lookouts, and other public spaces. We used a mix of “convenience sampling” and “quota sampling” (Bryman, 2012) in which we attempted to capture an approximately representative sample of people across demographic categories such as age, gender and income. A total of 1,934 local residents were surveyed, obtaining a response rate of over 50%. Not all questions were answered by all respondents. Residents were defined as people who live within the Reef catchment (East of Great Dividing Range, from Bundaberg to Cape York), while tourists lived anywhere outside of that area, either elsewhere in Australia or internationally (Marshall et al., 2016). All participants were over 18 years old and informed and verbal consent was obtained. Their information was recorded using iPads, and written consent would have been inappropriate and impractical.

### Data Analysis

In order to test our hypotheses, we fitted the value orientations and demographic factors (covariates) as fixed effects in multiple linear regression models in *R* (one for each climate change response: reef grief, environmental behavior and climate change beliefs). The significances of individual terms were tested at α = 0.05. The variance inflation factor was systematically smaller than 5, indicating low level of collinearity among covariates. Assumptions of linearity, homoscedasticity, and normality of residuals were checked by visual assessment of plots.

### A Description of the Sample Population

A description of the participants that agreed to partake in the study is presented in Tables 1, 2.

**Table 1:** A description of the survey population.

2017	GBR region coastal residents (*n* = 1934)
Mean age (±SE; range)	38.0 (± 0.37; 17–91)
Gender (F:M; %)	55:45
Years living in GBR region (±SE; range)	17.2 (±0.38; 1 month – 90 years)
Visited the GBR in lifetime?	94%
Visited the GBR in previous 12 months?	91%
Median household income (category)	$60,001–$100,000

**Table 2:** Results describing climate change beliefs at the global scale for residents of the Great Barrier Reef.

	% Residents (*n* = 1934)
Climate change is an immediate threat requiring immediate action	68.4
Climate change is a serious threat, but the impacts are too distant for immediate concern	13.2
I need more evidence to be convinced of the problem	11.8
I believe that climate change is not a threat at all	2.8
I do not have a view on climate change	3.8

## Results

### Value Orientations Within the Great Barrier Reef and Responses to Climate Change

The extent to which residents valued the GBR for biospheric, altruistic, egoistic and hedonic reasons are presented in Figure 1. Nearly 80% of coastal residents suggested that they would be personally affected if the health of the GBR declined, where over 37% provided 10 out of 10 for being affected (Figure 1). Some 37.6% of residents perceived that the coral reefs in their region were in good condition. Many respondents were not sure about coral reef condition, given that 18.4% recorded a 5/10 for their agreement with the statement, “the coral reefs in my region are in good condition” (Figure 1).

Nearly 82% of residents thought that global climate change was either an immediate or serious threat where over 68% of residents thought that climate change was an immediate threat requiring immediate action. Over 13% thought that climate change was a serious threat (Table 2). At a local scale, nearly 80% of residents were worried about climate change impacts on the GBR (Figure 1). A Pearson correlation analysis suggested that global and local perceptions of climate change were highly significantly correlated at the 0.01 level (2-tailed) (Pearson correlation = 0.285^∗∗^). Residents reported a mean level of Reef Grief of 7.14 on a scale of 1–10 (*SD* = 2.8).

### Hypotheses Testing

**FIGURE 2:**
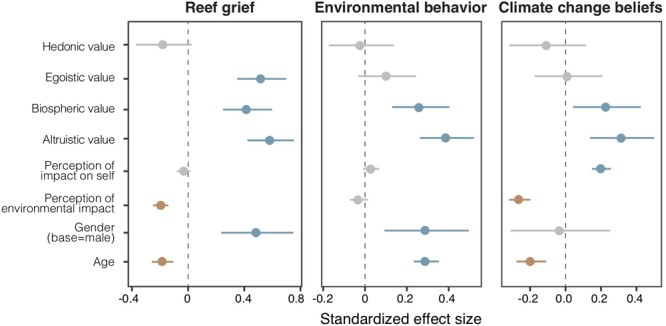
Regression plots showing the significance of cultural values, perceptions of impact (on self and environment), and demographic variables on each climate change response (reef grief, behavior, and beliefs).

Reef Grief was affected by three values (biospheric, altruistic, and egoistic), as well as by perception of environmental impact, and age and gender (Figure 2). Beliefs in climate change are influenced by two values (biospheric and altruistic), perceived personal impact, and perceived environmental impact, as well as age. Pro-environmental behaviors are influenced by two values (altruistic and biospheric), as well as age and gender (All climate change responses were highly significantly correlated with each other (*P* < 0.01) suggesting that people that believe in climate change are more likely to feel reef grief, and more likely to undertake pro-environmental behaviors).

## Discussion

Our work suggests that there is a strong internal relationship between the dimensions characterizing how people respond to change (beliefs, grief, and behavior). People that believe in climate change are more likely to feel reef grief, and more likely to undertake pro-environmental behaviors. Biospheric and altruistic values were important descriptors of each response. Egoistic values were important in describing reef grief. Age was also important in describing all responses, and gender was important to describe reef grief as well pro-environmental behaviors. Whether people perceived any environmental impact from climate change was correlated with reef grief and climate change beliefs. Whether people perceived that they would be affected by climate change was related only to their climate change beliefs.

An important tenet to this work is the observation that in 2013 51.7% of people believed that climate change was as an immediate threat requiring action (Marshall et al., 2016), whereas results from this study indicate that this percentage has increased significantly to 68.4%, suggesting that factors other than those measured in this study were important, or that the 2016 and 2017 bleaching events provided more awareness of the impacts of climate change for local residents. Given the importance of biospheric values in influencing how people respond to climate change, it is likely that people with biospheric values experienced, or were more interested in learning about climate change, where the events enabled a public discourse about environmental issues to occur. If so, then experiencing, or communicating about, climate change and its impacts can inspire behavior change in people with biospheric values, which were a very significant proportion of the local population in this study. Altruistic values, on the other hand, are perhaps more difficult to manage given that it unlikely that people might be persuaded to more highly rate intrinsic values (Howell and Allen, 2017). Perhaps though the use of communications that focuses on information and empathy (refs), it might be possible to inspire people to more highly value Traditional Owner heritage within the region and learn to develop altruistic values more broadly. Egoistic values, which were found here to be strongly correlated with reef grief, might be encouraged by communications that more broadly focus on the coastal hazard protection, health, lifestyle and wellbeing benefits associated with having natural resources such as the Great Barrier Reef, in great condition (Baldwin and Lammers, 2016). “Protect the Reef that protects us” campaigns would be powerful for those with high levels of egoistic values.

Regardless of any management intervention or communications, we can expect that, through time, the segment of society that sees climate change as an immediate threat requiring action will slowly grow. This momentum will, according to our results, occur as people more clearly experience local impacts and recognize environmental change. Older people and particularly conservative people, are unlikely to significantly contribute to society’s shift toward climate change acceptance and action in the near future, but it is possible if their environmental value-orientations are better understood (Goldberg J. et al., 2018; Goldberg J.A. et al., 2018). Marshall et al. (2018) recently showed that commercial fishers in the Great Barrier Reef, a typical conservative white male cohort, did in fact shift their perceptions of the urgency of climate change from 17% in 2013 to 26% in 2017 (Marshall et al., 2016, 2018). Targeting communication efforts toward the cultural values that people hold for natural resources is likely to be more effective in shifting people’s climate change attitudes and responses than trying to swing conservative white male denialism.

In sum, our hypotheses have been largely supported. Whilst we did not expressly set out to assess the influence of recognizing climate change impacts on the environment or on oneself, they both were important in influencing climate change response to some degree. We highlight that concepts such as grief were measured using only one survey question, and that a comprehensive insight into grief is only likely through more developed future work. Nonetheless, our work suggests that a significant phenomenon is likely to be at play, and more attention to ecological grief is warranted. Further, like above, communications that highlight the impact of climate change on important natural resources such as the Great Barrier Reef, the role of such resources in providing benefits to people, as well as inspiring people to be more empathetic, such as through valuing traditional owners, it may be possible to use cultural values such as biospheric, altruistic and egoistic values to shift people toward environmental stewardship and toward responses to climate change that are more adaptive and sustainable (Evans et al., 2013).

Responding effectively to climate change through understanding its urgency (beliefs) and impacts (environmental perceptions), feeling grief (that is altruistically or egoistically driven) and adopting appropriate pro-environmental behaviors, is critical for successfully meeting the future. The certainty of an altered world where wellbeing cannot necessarily be associated with natural resource condition, is already becoming apparent (Barnett et al., 2016). Yet, people value many things about natural resources, particularly iconic resources such as the GBR (Stoeckl et al., 2014; Esparon et al., 2015; Farr et al., 2016). Natural resources support identity, pride, place, esthetic appeal, biodiversity, lifestyle, heritage, and agency (Marshall et al., 2018). Accordingly, whilst ecosystems indeed contribute to making human life possible, they also contribute to making life worth living (Costanza et al., 1997). Using cultural values may be a useful way to communicate with people and to manage our natural resources effectively.

## Ethics Statement

This work received Ethics Approval from the CSIRO Social Science Human Research Ethics Committee 050/17.

## Author Contributions

All co-authors wrote the manuscript. NM collected the data and received the funds. LT, GG, and NM performed the data analysis.

## Conflict of Interest Statement

The authors declare that the research was conducted in the absence of any commercial or financial relationships that could be construed as a potential conflict of interest.
